# Characterization of the Antiglioma Effect of the Oncolytic Adenovirus VCN-01

**DOI:** 10.1371/journal.pone.0147211

**Published:** 2016-01-25

**Authors:** Beatriz Vera, Naiara Martínez-Vélez, Enric Xipell, Arlet Acanda de la Rocha, Ana Patiño-García, Javier Saez-Castresana, Marisol Gonzalez-Huarriz, Manel Cascallo, Ramón Alemany, Marta M. Alonso

**Affiliations:** 1 Navarra’s Health Research Institute (IDISNA) Pamplona, Spain; 2 Program in Solid Tumors and Biomarkers, Foundation for the Applied Medical Research, Pamplona, Spain; 3 Dpt of Medical Oncology, University Hospital of Navarra, Pamplona 31008, Spain; 4 Dpt of Pediatrics, University Hospital of Navarra, Pamplona 31008, Spain; 5 Brain Tumor Biology Unit, University of Navarra School of Sciences, 31008 Pamplona, Spain; 6 VCN Biosciences, Sant Cugat del Vallés, 08174 Barcelona, Spain; 7 Translational Research Laboratory, IDIBELL-Institut Catalá d’Oncologia, L’Hospitalet de Llobregat, 08907 Barcelona, Spain; University of Helsinki, FINLAND

## Abstract

Despite the recent advances in the development of antitumor therapies, the prognosis for patients with malignant gliomas remains dismal. Therapy with tumor-selective viruses is emerging as a treatment option for this devastating disease. In this study we characterize the anti-glioma effect of VCN-01, an improved hyaluronidase-armed pRB-pathway-selective oncolytic adenovirus that has proven safe and effective in the treatment of several solid tumors. VCN-01 displayed a significant cytotoxic effect on glioma cells *in vitro*. *In vivo*, in two different orthotopic glioma models, a single intra-tumoral administration of VCN-01 increased overall survival significantly and led to long-term survivors free of disease.

## Introduction

Glioblastoma multiforme (GBM) is the most common primary brain tumor in adults [1]. Multimodal treatment with surgery, radiotherapy, and chemotherapy extends mean survival by only a few months [2] whilst the overall survival after recurrence ranges from 3 to 9 months [3]. Therefore, the need for new therapeutic strategies is evident.

The use of oncolytic adenoviruses in tumour treatment is attractive because these viruses can be produced in high titers, do not integrate into the host chromosome, have a wide tropism, and possess a lytic life cycle that can be exploited for oncolysis [4]. Several selective oncolytic adenoviruses have shown promising results in the treatment of GBM in the preclinical setting [5–8]. However, these viruses encounter a major hurdle which is the distribution within the tumor due to physical limitations. They face transport barriers in the tumor interstitium, due to their size (90–100 nm), much larger than chemotherapeutic drugs and that impairs optimal distribution in solid tumors. The main sources of physical resistance to virus (and drug) transport are the extracellular matrix (ECM) and interstitial fluid, both of which are abundant in solid tumors. Of relevance for this work, the high-molecular-weight glycosaminoglycan, hyaluronic acid (HA), makes up a significant portion of the brain extracellular matrix [9,10]

The current study investigates the antiglioma effect adenovirus VCN-01, which is a modified virus based on the ICOVIR platform [11]. We previously showed that ICOVIR-5, a previous version of VCN-01, in which the control of E1A is under an E2F-1 promoter insulated with the myotonic dystrophy locus insulator showed a safe toxicity profile and a robust antiglioma effect [12,13]. The modifications incorporated in VCN-01 are; a selective replication depending on pRB pathway deregulation (for tumor selectivity), a retargeting modification RGDK in the fiber shaft binding (for tumor targeting) and the expression of a modified hyaluronidase (to break down the extracellular matrix). Previously, it has been shown that VCN-01 induces a potent and selective replication profile when delivered systemically, and they demonstrate that the modifications described above improved antitumor efficacy while maintaining a safe toxicity profile [14]. In this work we seek to characterize the antiglioma effect in vivo and in vitro of the oncolytic adenovirus VCN-01. VCN-01 showed a potent oncolytic effect in vitro against a panel of cell lines. Of importance, treatment of two different in vivo orthotopic glioma models with VCN-01 resulted in a significant increase in overall survival and in the number of long-term survivors. Overall, these results encourage further development of VCN-01 against malignant gliomas.

## Materials and Methods

### Cell lines and culture conditions

Glioma cell lines U87MG, A172, T98G, U251MG, U373MG and SNB19 were obtained from the American Type Culture Collection (Manassas, VA, USA). Cell lines were maintained in DMEM/F12 (1:1, v/v) (Gibco, Life Technologies, NY, USA) supplemented with 10% fetal bovine serum in a humidified atmosphere containing 5% CO_2_ at 37°C. The adult glioma brain tumor stem cell (BTSC lines GSC23, GSC11 were kindly provided by Dr. Lang (Department of Neurosurgery; MD Anderson Cancer Center, USA). BTSCs were cultured as neurospheres. The neurospheres were maintained in Dulbecco's modified Eagle/F12 medium (1:1, vol/vol) (Thermo Fisher Scientific Inc, Waltham, MA) supplemented with B27 (Thermo Fisher Scientific Inc, Waltham, MA), basic Fibroblast Growth Factor and Epidermal Growth Factor (20 ng/mL Sigma-Aldrich, St Louis, MO).

### Cell viability assay

Cells were seeded in 96-well plates at a density of 1x10^3^ cells per well. Cells were infected with VCN-01 on the same day as seeding. VCN-01 construction was carried out as described previously [14]. MOI ranged from 1 to 250. Cell viability was assessed five days later using the MTT assay (Sigma-Aldrich, St. Louis, MO, USA) as described by Mosmann [15]. Dose–response curves were analyzed using CalcuSyn Software (Biosoft, Cambridge, UK). IC50 is the median-effective dose, that is, the dose that causes 50% of cells to be affected, which, in this case, is equivalent to the dose that results in 50% survival.

### Quantitative RT-PCR of mRNA levels

Quantitative RT-PCR was performed with ABI Prism-7500 (Applied Biosystems, Foster City, CA, USA). The primers used for detection of hyaluronidase PH20 and Fiber mRNA transcripts were:

- PH20 forward primer: 5′- CTTAGTCTCACAGAGGCCAC-3′
- PH20 reverse primer: 5′- CCAGGAGCAAGGAGTGTGTA-3′
- Fiber forward primer: 5′- ATTTGCCACATCCTCTTACAC -3′
- Fiber reverse primer: 5′ CAAACGCTGTTGGATTTATG -3′

The cycling conditions for PCR were one cycle of 10 minutes at 95°C followed by 15s at 95°C, 1 minute at 60°C for 40 cycles. To determine relative gene expression, the comparative threshold cycle (*C*_T_) method was used [16].

### Western Blot analysis

Whole-cell lysate protein samples were resolved by SDS-PAGE, electro-blotted to a nitrocellulose membrane (Bio-Rad, Hercules, CA, USA) and incubated with the following antibodies: rabbit anti-E1A (Santa Cruz Biotechnology, Santa Cruz, CA, USA) and mouse anti-Fiber (Thermo Scientific, MA, USA) and GRB2 (Santa Cruz Biotechnology, CA, USA).

### Viral replication assays

Cell cultures were grown to 60–80% confluence in 24-well plates and subsequently infected at an MOI that allowed at least 80% infectivity. On day 4 after infection cells and medium were harvested and freeze–thawed three times. Viral titers in cell extracts were determined according to an anti-hexon staining-based method [17].

### Animal studies

In order to assess the antiglioma effect of VCN-01 *in vivo* we used two different glioma mouse models. The first one is the well-established U87 MG model. The other model uses the BTSC GSC23 cell line, which develops infiltrative and diffuse tumors that reflect the phenotype of gliomas in human patients.

Ethical approval for animal studies was granted by the Animal Ethical Committee of the University of Navarra (CEEA; Comité Etico de Experimentación Animal under the protocol number CEEA/069-13). All animal studies were done in the veterinary facilities of the Center for Applied Medical Research in accordance with institutional, regional, and national laws and ethical guidelines for experimental animal care. The animals were monitored on daily basis and were euthanized when they demonstrate moribund behavior including: slight head tilt, hemiparesis, hunched posture, scleral edema, inability to access food/water, weight loss >20% of baseline, and excessive tumor burden as indicated by doming of cranium >0.5 cm, or if show signs of lower extremity weakness. The animals were sacrificed with CO2 inhalation. To minimize suffering of the animals, ketamine/xylazine or buprenorphine was given for signs of pain, eye wincing, hunched state with front limbs over the head.

Nude (*nu/nu*) mice were obtained from Taconic Farms Inc. U87 MG (5 × 10^5^) and GSC23 cells (3 × 10^5^) were engrafted into the caudate nucleus of athymic mice by means of the guide-screw system described by [18]. On the third day after implantation of tumour cells, tumours were injected with 5-μL of PBS, VCN-01 inactivated with UV light or VCN-01 solution: 10 mice received PBS, 10 mice received VCN-01 at 1×10^8^ pfu/mouse previously inactivated with UV (UVi), 10 mice received VCN-01 at a concentration of 1 × 10^7^ pfu/mouse, and 10 mice received VCN-01 at 1×10^8^ pfu/mouse).

### Immunohistochemical analysis

Tumor xenografts and paraffin-embedded sections of mouse tumors were treated goat anti-hexon antibody (Chemicon, CA, USA). For immunohistochemical staining, Vectastain ABC kits (Vector Laboratories, CA, USA) were used according to the manufacturer's instructions.

### Statistical analysis

For the *in vitro* experiments, data are expressed as mean ± SD, and comparisons were by the two-tailed Student *t* test. The *in vivo* cytopathic effect of VCN-01 on human glioma xenografts was assessed by plotting survival curves according to the Kaplan-Meier method. Survival in different treatment groups was compared using the log-rank test.

## Results

### VCN-01 exerts a potent antitumor effect in established and brain tumor stem cell lines

**Fig 1:**
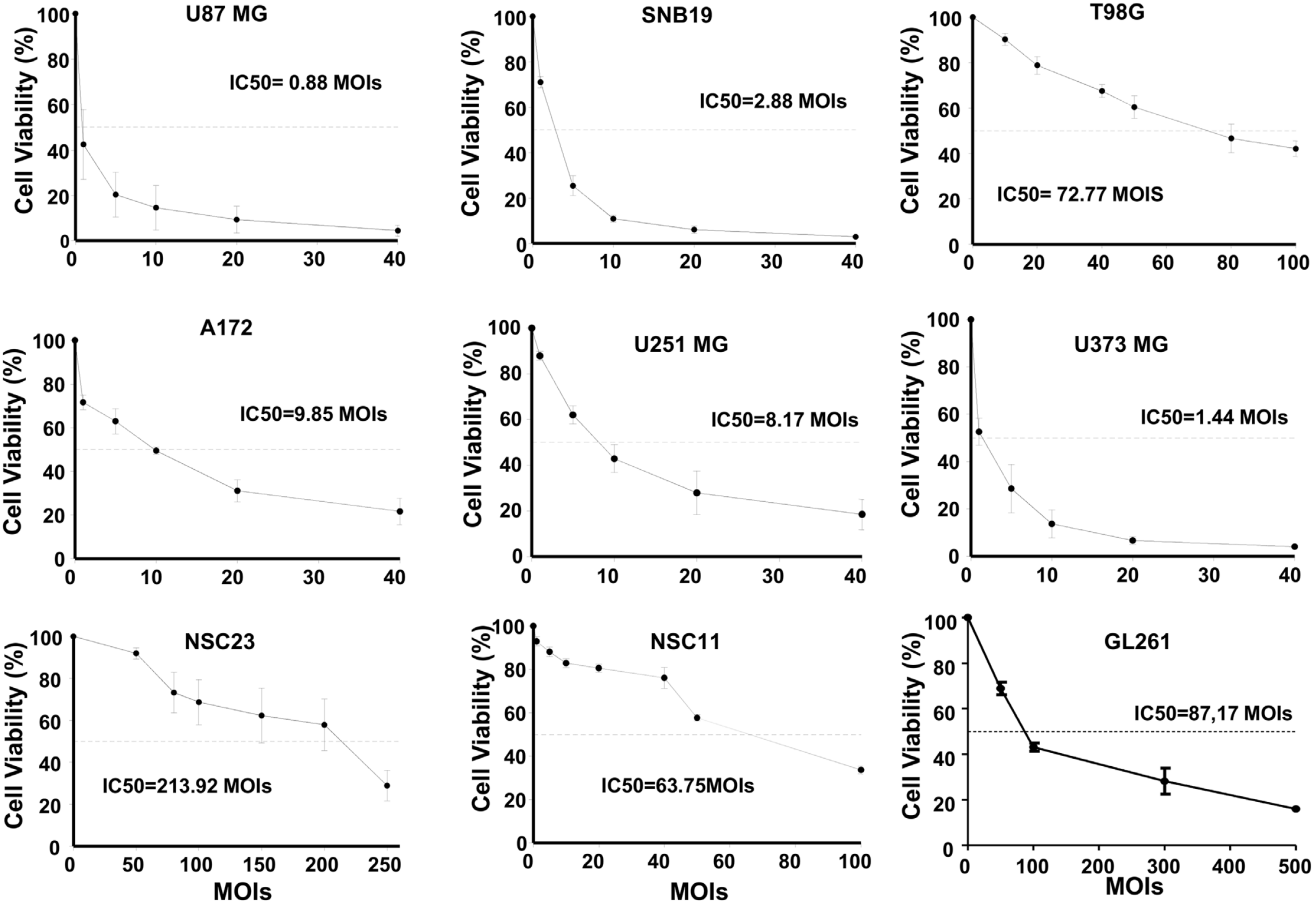
VCN-01 exerts a potent antitumor effect in established and brain tumor stem cell lines. (A) Cell viability analyses of VCN-01 infected glioma cell lines. Cell viability was assessed using 3-(4,5-dimethylthiazol-2-yl)-2,5-diphenyltetrazolium bromide (MTT) assays five days after infection. Data are shown as the percentage (mean ±SD) of cells alive after infection with VCN-01 at the indicated multiplicities of infection (MOIs) relative to cells infected with UV-inactivated VCN-01 (control, equal to 100%).

To examine the antiglioma effect of the oncolytic adenovirus VCN-01 we performed viability assays in a battery of established glioma cell lines (U87-MG, A172, T98G, U251-MG, U373, and SNB19) and brain tumor stem cells (BTSC, GSC23, and NSC11) and also in the murine glioma GL261. We choose the GL261 as a possible resistant cell line since adenovirus replication is compromised in murine cell lines. All the glioma cell lines tested were susceptible to the cytotoxic effect of VCN-01. MOI IC_50_ ranged from 0.88±0.12 to 213±18. U87-MG was the most sensitive cell line, and GSC23 the most resistant (Fig 1). As expected BTSC lines were more resistant than established glioma cell lines at a similar or higher level than the virus resistant GL261 cell line. Dose-response cytotoxicity was observed in all cell lines.

### VCN-01 replicates efficiently in vitro

**Fig 2:**
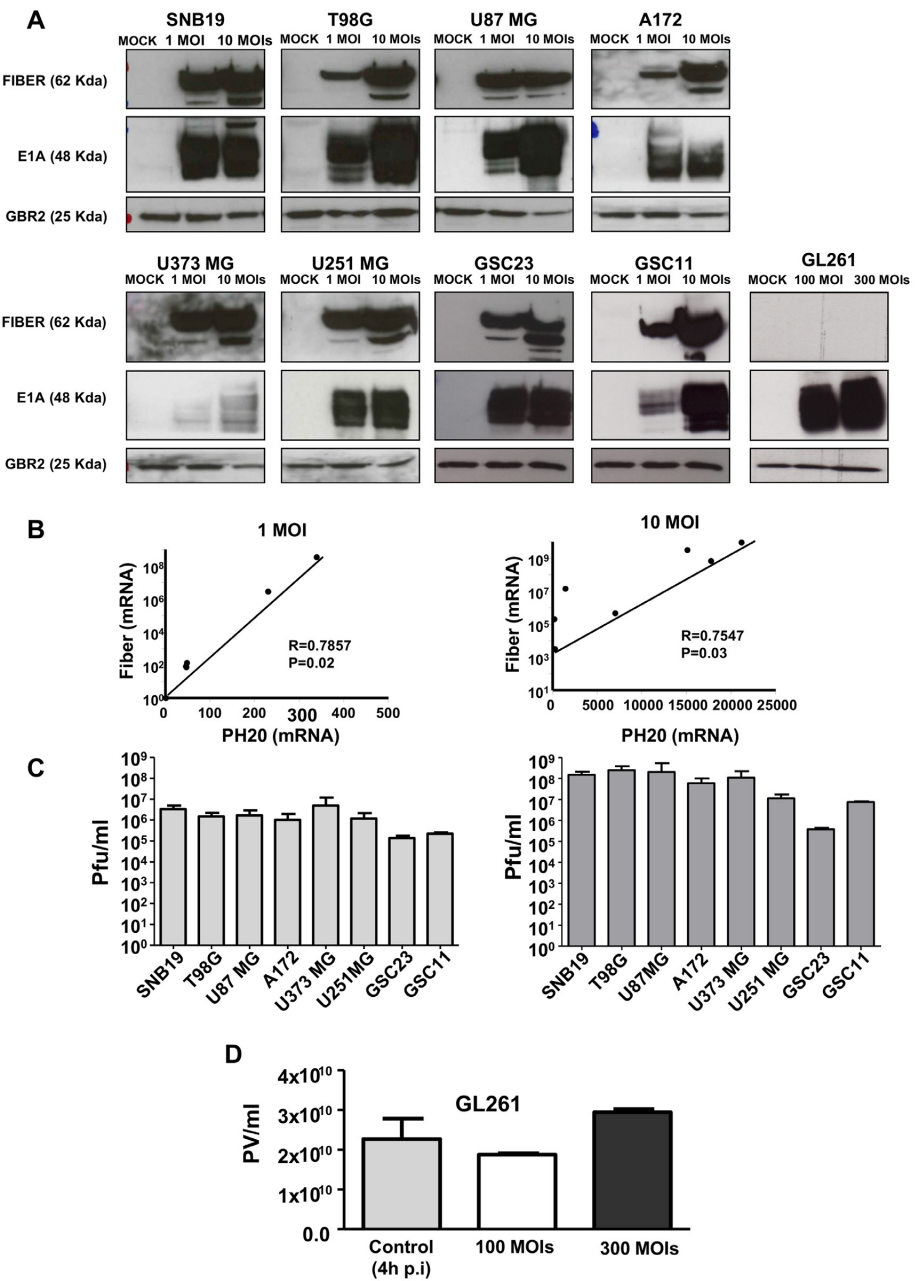
VCN-01 replicates efficiently in glioma cell lines *in vitro*. (A) The expression amounts of Fiber and E1A proteins in human glioblastoma were determined by Western blot analysis. Note that the levels were viral dose-dependent in all the cell lines. (B) The expression of Fiber and PH20 mRNA in all the cell lines were determined by real time PCR; expression of both genes was correlated, suggesting similar kinetics and dose-dependency. The cell lines used were SNB19, T98G, U87 MG, A172, U373 MG, U251 MG, GSC23 and GSC11. (C) The quantification of VCN-01 viral replication in all the cell lines. Human glioblastoma cells were infected with two dilutions of VCN-01 (1 MOI and 10 MOIs). The virus replicated up to ten-times better in the cells infected with 10 MOIs, which demonstrates that the virus infected and replicated efficiently in glioblastoma cell lines *in vitro*. (D) The quantification of VCN-01 viral replication in GL261 murine cell line. GL261 cells were infected with two dilutions of VCN-01 (100 MOI and 300 MOIs). As a control for the replication we used cells infected with VCN-01 at 300 MOIs and collected 4 hours after infection, during this time the virus does not have time to replicate indicating the initial viral particles.

Replication was assessed by characterization of the expression of early and late adenoviral proteins. E1A is expressed immediately after infection and initiates the viral replication cycle [19]. Fiber is a protein component of the capsid, it is expressed late in the replication process, and it is thereby indicative of an effective viral replication cycle. Cells were infected at either 1 or 10 MOIs and protein expression was assessed 48 hours later. We observed a robust and dose dependent expression of both E1A and Fiber proteins in all the cell line tested (Fig 2A) except in the GL261 cell line where, as expected, fiber expression was absent. In addition, we observed E1A and Fiber expression differences amongst the cell lines used suggesting differences in the susceptibility to the virus effect. Next, we were interested in assess whether the hyaluronidase (PH20) protein was being efficiently expressed by VCN-01. Analyses of PH20 mRNA expression in all the cell lines used in this study (except GL261) showed a consistent mRNA expression that was virus-dose dependent (Fig 2B). The expression of PH20 mRNA correlated with the expression of Fiber (Fig 2B), suggesting similar kinetics.

Finally, quantification of VCN-01 replication and comparison of replication in cells infected with 1 MOI or 10 MOI indicated that VCN-01 replicated effectively in all the cell lines tested (Fig 2C). Replication was less effective in the BTSC lines, GSC23 and GSC11. Viral replication was up to ten-fold higher in cells infected at a MOI of ten as compared to those at a MOI of one. We also assessed the replication of VCN-01 in GL261 cell line and as expected we did not observed any replication even at the highest dose used (300 MOIs) when compared with the control (VCN-01 infected cells at 300 MOIs at 4 hours) (Fig 2D).

In conjunction, these results indicate that the virus infects and replicates effectively in glioma cell lines. Moreover, *in vitro*, the virus expresses PH-20.

### VCN-01 treatment results in a significant antitumor effect in two orthotopic glioma models

**Fig 3:**
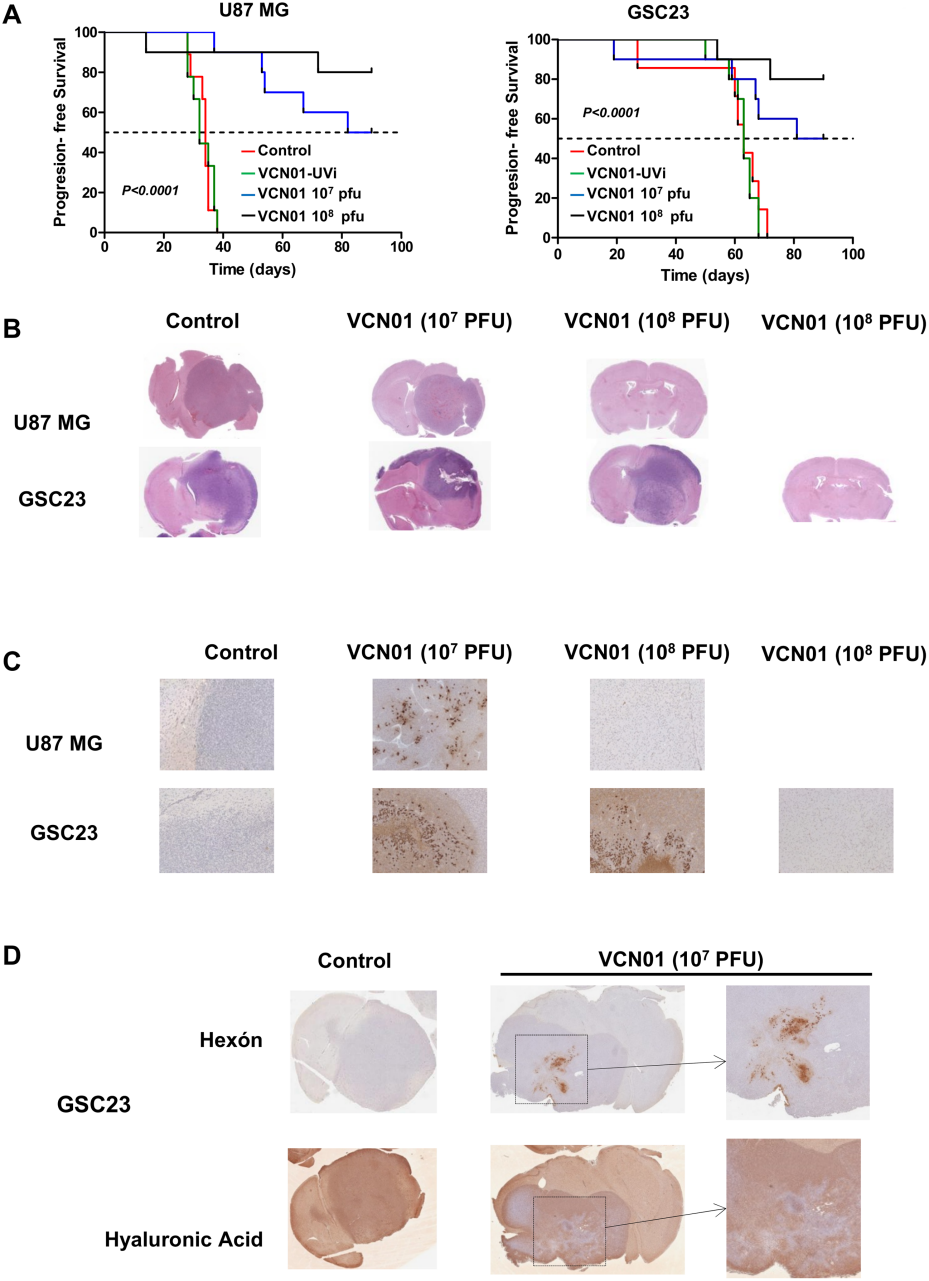
VCN-01 treatment results in a significant antitumor effect in two different *in vivo* glioma models. (A) Kaplan-Meier survival curves for overall survival in VCN-01 (10^7^ pfu), VCN (10^8^ pfu) and control (PBS)-treated athymic mice bearing U87 MG and GSC23 intracranial xenografts. Intracranial implantation of U87 MG or GSC23 cells (day 0) was followed by one intratumoral injection (on day 3) of VCN-01 (10^7^ pfu; *n* = 10), VCN-01 (10^8^ pfu; *n* = 10), VCN-01 10^8^ pfu UV inactivated (VCN01-UVi; *n* = 10) or PBS (*control*; *n* = 10). The *P* values were determined by the log-rank test and represent a comparison of survival of VCN-01–treated mice with that of mice treated with PBS. (B) Hematoxilin and eosin staining of cross sections of U87 MG or GSC23 xenografts treated with PBS or VCN-01. (C) Hexon immunostaining of the brains of animals treated with VCN-01 (10^7^ pfu), VCN (10^8^ pfu) or control (PBS). The tissue sections were incubated with anti-hexon antibodies. The magnification of histo-chemical images is ×20. (D) Representative Hyaluronic acid and hexon immunostaining of the brains of animals bearing GSC23 cell line treated with VCN-01 (10^7^ pfu), or control (PBS). The tissue sections were incubated with anti-hyaluronic acid and hexon antibodies. The magnification of histo-chemical images is ×20.

Mice bearing U87 MG or GSC23 intracranial xenografts were given intratumorally a single injection of PBS (control), VCN-01 inactivated with UV (VCN-UVi 1x 10^8^ pfu/mouse) VCN-01 (1x10^7^ pfu/mouse) or VCN-01 (1x 10^8^ pfu/mouse). The median survival for mice that received PBS or the virus inactivated with UV was 34/32 and 63 days, for U87 MG and NSC-23 mice, respectively. In contrast, the median survival of mice treated with VCN-01 at 1x10^7^ pfu were 86 and 85.5 days, for U87 MG and GSC23 mice, respectively. All U87-MG mice injected with PBS or UV-inactivated virus died by day 39, and all GSC23 mice injected with PBS or UV-inactivated virus died by day 70. Meanwhile, 80% of mice (both models pooled) whose tumours were injected with 1x10^8^ pfu of VCN-01 were alive at 90 days (Fig 3A).

Hematoxylin and eosin (H&E) staining revealed large, aggressive tumours in PBS control mice while mice that received 10^7^ pfu VCN-01 had smaller tumours with extensive necrotic areas surrounded by areas displaying prominent viral inclusion bodies suggestive of effective viral infection and replication (Fig 3B). These latter areas typically stained for hexon proteins (Fig 3C). Immunohistochemical studies of the brains of long-term survivors (of both U87 MG and GSC-23 mice) showed no evidence of residual tumor; there was no staining with anti-hexon antibodies, indicating an absence of viral particles (Fig 3C). In addition, we assessed the expression of hyaluronic acid in the different treatment groups and compare it with the expression of hexon. Brains from control animals showed a uniform hyaluronic acid staining throughout the tumor and the brain. Meanwhile in VCN-01 treated animals we could observed that in areas surrounding hexon staining there was less hyaluronic acid staining suggesting the effect of the hyaluronidase enzyme (Fig 3D; representative images from GSC23 bearing mice).

In summary, a single intra-tumoral injection of VCN-01 resulted in significant improvement in survival in two different mouse models for glioma. These data suggest that VCN-01 may be a promising therapeutic agent for glioma.

## Discussion

GBM is an aggressive tumor whose progression current therapies only manage to delay by a few months. In the preclinical glioma study reported here, we characterize the anti-tumor effect of VCN-01, a new generation oncolytic adenovirus developed by genetic modification from the ICOVIR platform [14]. We found VCN-01 to induce a potent antitumor effect *in vitro* and *in vivo*, and we therefore consider this virus to be a promising potential treatment for gliomas.

VCN-01 replication is engineered to require the existence of free E2F and is therefore dependent on pRB pathway deregulation. Overexpression of E2F1 protein and the activation of E2F-responsive promoters have been reported, by our group and others, to occur in gliomas [20,21] and consequently, VCN-01 replication is expected to proceed in glioma cells. This expectation was confirmed in the current study.

Several years ago our group published results of a study into the antiglioma effect of a precursor of VCN-01, ICOVIR-5, which combines E1a transcriptional control by an insulated form of the E2F promoter with the Delta24 mutation of E1a. ICOVIR-5 has also the Kozak sequence at the E1a start codon, and this sequence is important to restore E1a expression and viral replication to AdwtRGD levels in tumor cells. ICOVIR-5 proved to be a highly selective vector in glioblastoma preclinical models [12] and non-toxic to the model animal when administered systemically [13]. The drawback, however, was that ICOVIR-5 was less potent than the same adenovirus without the E2F promoter. In contrast, VCN-01 is not only a safe vector when delivered systemically but has a significant antitumoral effect in different solid tumors [14].

In the current *in vivo* study, we used two different tumour models: a U87MG-based model, which is aggressive but does not reflect the infiltrative phenotype of GBM; and a GSC23 model, which is a BTSC line and gives rise to extremely aggressive and invasive tumours. Even with GSC23-derived tumours, VCN-01 demonstrated effective antitumor activity with a single injection.

Our study does not compare VCN-01 with other viruses, such as ICOVIR-5, and therefore it remains a matter of speculation whether the enhanced antitumor effect is due to the improved infectivity plus the expression of a soluble hyaluronidase (PH20). Another consideration not covered by our study is the effect of VCN-01 in an immunocompetent model. It has been speculated that the antitumor effect of oncolytic viruses is due not only to the intrinsic cytolytic effect of the virus but also to the triggering of an immune response against the virus and in turn against the tumoral cells hosting that virus. In this respect, it is interesting to note that hyaluronidase is known to be immunogenic [22–26].

In conclusion, *in vitro*, VCN-01 effectively infected cells and replicated in glioma cell lines, significantly reducing tumor growth. *In vivo*, a single intra-tumoral injection of VCN-01 resulted in significant (*P*<0.0001) improvement in survival in two different mouse models for glioma. These preclinical results suggest that VCN-01 warrants further study to evaluate its potential in the treatment of malignant gliomas.
