# Correction: Characterization of the Antiglioma Effect of the Oncolytic Adenovirus VCN-01

**DOI:** 10.1371/journal.pone.0157619

**Published:** 2016-06-09

**Authors:** Beatriz Vera, Naiara Martínez-Vélez, Enric Xipell, Arlet Acanda de la Rocha, Ana Patiño-García, Javier S. Castresana, Marisol Gonzalez-Huarriz, Manel Cascallo, Ramón Alemany, Marta M. Alonso

The sixth author’s name is misspelled. The correct name is: Javier S. Castresana. The correct citation is: Vera B, Martínez-Vélez N, Xipell E, Acanda de la Rocha A, Patiño-García A, Castresana JS, et al. (2016) Characterization of the Antiglioma Effect of the Oncolytic Adenovirus VCN-01. PLoS ONE 11(1): e0147211. doi:10.1371/journal.pone.0147211
